# Meckel’s Diverticulum in Crohn’s Disease Revisited: A Case of Meckel’s Diverticulitis in a Patient with Stricturing Crohn’s Disease

**DOI:** 10.7759/cureus.2865

**Published:** 2018-06-22

**Authors:** Thamer Kassim, Abdullah Abdussalam, Erin Jenkins

**Affiliations:** 1 Internal Medicine, Creighton University Medical Center, Omaha, USA; 2 Gastroenterology, Creighton University, Omaha, USA

**Keywords:** meckel's diverticulum, meckel's diverticulitis, meckel's diverticulum in crohns disease, crohns disease, stricturing crohns disease

## Abstract

According to anecdotal reports in literature, encountering Meckel’s diverticulum in a patient with Crohn’s disease is not uncommon, but differentiating between the overlapping complications of Mickel’s diverticulum and the natural manifestations of Crohn’s disease can be challenging and may impact lifelong therapy. In this report, we present a case of Meckel’s diverticulitis in a patient with stricturing ileocolonic Crohn’s disease. A 29-year-old male has been suffering from recurrent bouts of abdominal pain and diarrhea which were initially thought to be due to recurrent flares of Crohn’s disease. The patient was started on different medical regimens to control his disease, but complete remission was not achieved. He was found to have an inflamed Meckel’s diverticulum during laparotomy with sections of transmural inflammation extending into the diverticulum with absence of heterotopic mucosa. Although Meckel’s diverticulum and Crohn’s disease involve separate disease processes and different pathogenesis, several hypotheses to explain a correlation have been suggested. We believe it is important to consider the presence of an inflamed Meckel’s diverticulum in the differential diagnosis for patients with refractory Crohn’s disease who do not have an adequate response to medical therapy.

## Introduction

The prevalence of Meckel’s Diverticulum (MD) in patients with Crohn’s Disease (CD) remains unclear. MD and CD can have overlapping symptoms, yet their underlying pathophysiology is different. The association between MD and CD is not very well understood, but differentiating between both disease processes can have a significant impact on lifelong therapy. We present a case of Meckel’s diverticulitis with absence of heterotopic mucosa in a patient with stricturing ileocolonic CD.

## Case presentation

A 29-year-old male patient known to have a history of gastroesophageal reflux disease and polysubstance abuse presented to the emergency department complaining of peri-umbilical abdominal pain, diarrhea, bright red bleeding per rectum, and dizziness. The patient had been suffering from similar symptoms episodically for the past 15 years. Previous abdomen computed tomography (CT) scan without contrast at age of 23 showed cecal thickening, after which the patient was treated with ciprofloxacin and metronidazole with minimal improvement. Subsequently, the patient was admitted as a case of suspected inflammatory bowel disease (IBD).

On physical examination, vital signs were: blood pressure 155/83 mmHg, heart rate 99 beats per minute, temperature 98.8 F, and respiratory rate 16. The patient appeared pale. Abdominal exam revealed normoactive bowel sounds, right lower quadrant tenderness, and no organomegaly. Physical exam was unremarkable otherwise. Laboratory workup was remarkable for iron deficiency anemia (Table 1).

**Table 1:** Laboratory tests consistent with iron deficiency anemia.

Laboratory test	Laboratory value	Reference range
Hemoglobin (gm/dl)	6.9	13.5-17.5
Mean corpuscular volume (fl)	60	80-100
Mean corpuscular hemoglobin (pg)	16.7	26-34
Red cell distribution width (%)	20	11.5 – 15
Serum Iron (ug/dl)	10	49-181
Ferritin (ug/dl)	2	49-181
Total iron binding capacity (ug/dl)	467	250-450

Fecal calprotectin was elevated at 90 μg/g (reference range: <51 μg/g), and C-reactive protein (CRP) and erythrocyte sedimentation rate (ESR) were within reference range. Comprehensive metabolic panel, celiac disease panel, lipase, amylase, and stool studies, including Clostridium difficile toxin, were within normal limits.

Abdomen CT with contrast showed mesenteric lymphadenopathy with no findings of bowel thickening. Magnetic resonance enterography (MRE) showed a dilated segment of the small bowel with a possible diverticulum. The gastroenterology service was consulted with suspicion for IBD, in particular ileocolonic CD causing a stricture. Esophagogastroduodenoscopy and ileocolonoscopy were performed, which showed healthy mucosa of the colon and ileum with no endoscopic changes suggestive of IBD. Random ileal and colonic biopsies were obtained, and pathology of colonic biopsies revealed mild active chronic colitis with focal cryptitis. Ileal biopsies showed mild mucosal lymphoid hyperplasia. At discharge, adalimumab was initiated at standard dose for the possible stricturing ileocolonic CD. The patient was later readmitted with similar symptoms.

**Figure 1:**
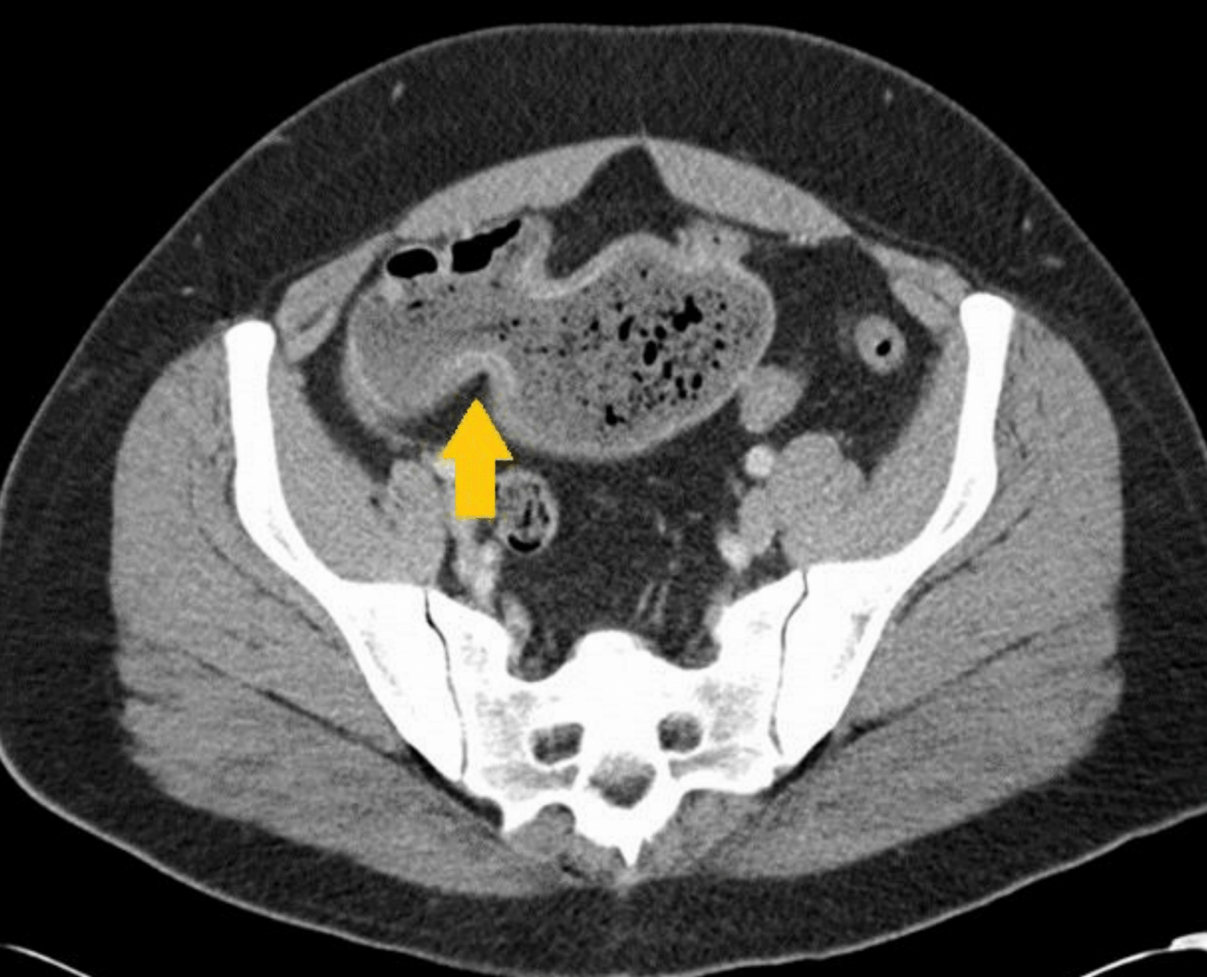
Contrast enhanced computed tomography (CT) abdomen/pelvis showing a segment of small bowel in the right lower quadrant with areas of stricture and dilation.

During his second admission, CRP was within normal limits. Abdomen CT scan with contrast and MRE showed small bowel wall thickening and inflammation within a bowel loop in the right lower quadrant with partial obstruction and dilation of involved loop (Figure 1).

**Figure 2:**
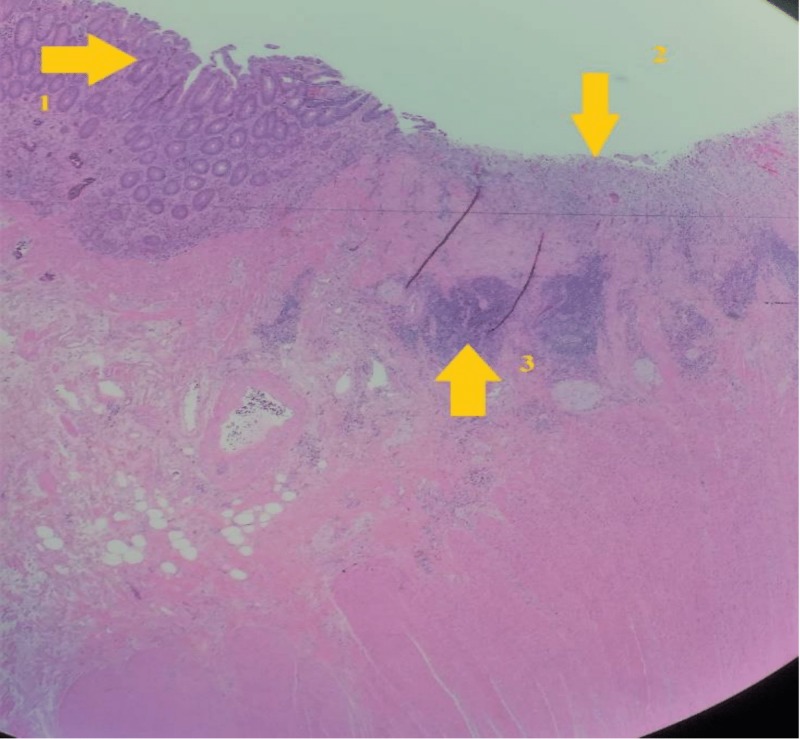
Section of Meckel’s Diverticulum showing areas of transmural inflammation extending into the diverticulum mucosa. 1. Normal Mucosa 2. Mucosal ulceration 3. Inflammatory cells spreading transmurally.

Esophagogastroduodenoscopy and ileocolonoscopy were repeated, and again, there were no endoscopic changes suggestive of active disease in the colon or terminal ileum. Biopsies showed normal ileal and colonic mucosa. The colorectal surgery service was consulted due to the possibility that these findings may be due to regional enteritis from MD rather than active CD, and resection of the affected small bowel segment was recommended. The patient underwent the surgery three months later. During laparotomy, there was a tight stricture two feet away from the ileocecal valve with outpouching of a thickened segment of bowel suggestive of MD. Pathology showed a two-inch wide diverticulum with sections of transmural inflammation and aphthous ulceration. No gastric or pancreatic mucosa were seen (Figure 2).

The patient was continued on adalimumab and followed up later to report resolution of symptoms. We hypothesize that our patient had underlying ileocolonic Crohn’s disease, but with symptoms related to Meckel’s diverticulitis.

## Discussion

MD is a congenital anomaly resulting from incomplete obliteration of the vitelline duct, which consequently leads to the formation of a true diverticulum of the small intestine. It is the most common congenital malformation of the gastrointestinal tract. The incidence of MD is estimated to be 2% of the general population [1].

Multiple studies have been done to assess the prevalence of MD in CD, and it was suggested by Andreyev et al., in their review of 294 patient with CD, that the prevalence of MD in patients with CD is three times above that of the general population [2]. Andreyev’s study results correlated with previous anecdotal reports by Bondeson and Starck-Bondeson [3] who found 13 Meckel’s diverticula out of 216 patients with CD (6%) and Ekman [4] who found 5/27 (18.5%). However, these results were challenged by Freeman [5] who found only 10 of 877 patients with CD had MD (approximately 1%) and argued that MD prevalence in patients with CD is actually similar to that of the general population.

As CD remains a disease of uncertain etiology, several hypotheses to explain a correlation between MD and CD have been suggested. Some have postulated that CD may be secondary to a developmental anomaly of lymphatic drainage, as congenital anomalies are often multiple. Another hypothesis is that the diverticulum serves as a reservoir for microbial organisms, increasing the probability of exposure to the organism responsible in CD. The gastrointestinal microbiota is gaining increasing recognition as a possible factor in the etiology of Crohn’s disease making this hypothesis plausible [3]. It is reasonable to question how two different diseases with two different pathogenesis, one congenital and the other multifactorial, are related. Therefore, it is also reasonable to think that these may be simultaneous but unrelated processes occurring in the same patient. Nevertheless, differentiating between both disease processes is vital as it can change the course of the patient’s management and impact lifelong therapy.

About 50% of patients with MD have heterotopic mucosa, gastric being the most common accounting for 65%. Other types of heterotrophic mucosa within MD are pancreatic and rarely colonic [2]. Chronic gastric secretions from heterotopic ectopic mucosa can rarely result in terminal ileum inflammation, mimicking terminal ileitis caused by CD, but histological changes should differentiate between them. Also, such patients generally have milder symptoms that do not warrant surgical intervention. As 50% of patients lack heterotopic gastric mucosa, pre-operative diagnosis of MD by technetium-99m pertechnetate scan remains very difficult, particularly in this patient population, since the test carries a high false positive rate in patients with IBD [1-2,5].

## Conclusions

Both MD and CD have similar overlapping symptoms and radiographic findings, which makes it difficult to differentiate one from the other. Meckel’s diverticulum can be a mimicker of Crohn’s disease as both diseases can present with recurrent cramping abdominal pain, bright red blood per rectum, and diarrhea. In our case, the patient’s symptoms were initially attributed to CD, and therapy was initiated without improvement until undergoing small bowel resection. It is clinically challenging to understand the basis of symptoms in such a patient population as we encounter many patients with refractory CD who do not have an adequate response to medical therapy. As clinical providers, we must consider that such patients may have another active underlying disease process contributing to the clinical symptoms.
